# Microfiber emission from a municipal wastewater treatment plant in Hungary

**DOI:** 10.1038/s41598-024-62817-2

**Published:** 2024-05-27

**Authors:** Davaakhuu Tserendorj, Ádám Illés, Ágnes Károly, Rita Stadler-Szalai, Sirat Sandil, Tamás Mireisz, Péter Dobosy, Flóra Pomázi, Sándor Baranya, Mónika Adányi, Gyula Záray

**Affiliations:** 1 Institute of Aquatic Ecology, HUN-REN Centre for Ecological Research, Karolina Út 29-31, Budapest, 1113 Hungary; 2 National Laboratory for Water Science and Water Security, Institute of Aquatic Ecology, HUN-REN Centre for Ecological Research, Karolina Út 29-31, Budapest, 1113 Hungary; 3 Hungarian Institute for Forensic Sciences, Mosonyi Str. 9, Budapest, 1087 Hungary; 4 Doctoral School of Environmental Sciences, Eötvös Loránd University, Pázmány Péter Sétány 1/C, Budapest, 1113 Hungary; 5 Department of Hydraulic and Water Resources Engineering, Faculty of Civil Engineering, Budapest University of Technology and Economics, Műegyetem Rkp. 3, Budapest, 1111 Hungary; 6 National Laboratory for Water Science and Water Security, Faculty of Civil Engineering, Department of Hydraulic and Water Resources Engineering, Budapest University of Technology and Economics, Műegyetem Rkp. 3, Budapest, 1111 Hungary; 7 Institute of Chemistry, Eötvös Loránd University, Pázmány Péter Sétány 1/A, Budapest, 1113 Hungary

**Keywords:** Environmental chemistry, Environmental impact

## Abstract

Since the ingestion of both natural and anthropogenic microfibers produces a deleterious effect on aquatic organisms, it is crucial to explore the emission of these pollutants by WWTPs into the receiving water bodies, such as rivers. Cellulose- and petroleum-based microfibers, as well as microplastic particles, were collected from the effluent of a municipal WWTP operating with activated sludge technology in Budapest, Hungary. During two sampling campaigns organized in February and April of 2023 on different working days and at different times of the day, 123–145 L of effluent was sieved and filtered. The organic matter was removed by hydrogen-peroxide treatment. All fibers and particles larger than 10 µm were counted, and using a fluorescence microscope, the fibers were geometrically characterized in terms of length and diameter. Each fiber was individually identified by transflection-FT-IR method. The fiber concentration varied in the range of 1.88–2.84 and 4.25–6.79 items/L during the 7th and the 16th week of 2023, respectively. In February and April, the proportion of microfibers in the solid particles was 78.3 and 94.7%, respectively. In the effluent the cellulose-based microfibers were dominant (53–91%), while among the petroleum-based microfibers, polyester occurred most often. The median length of cellulose-based fibers was considerably higher in April than in February (650 vs. 1250 µm), and simultaneously the median diameter also increased from 21 to 29 µm. This behaviour was also seen, albeit to a lesser extent, in connection to microfibers derived from petroleum. The treated wastewater’s daily microfiber transport to the Danube River varied between 0.44 − 0.69 and 0.94–1.53 billion in February and April 2023, respectively.

## Introduction

The effluents of municipal wastewater treatment plants (WWTPs) located along riverbanks are the main sources of various solid particles (fibers, fragments, and pellets) influencing the aquatic life in the riverine environment^1–9^. Due to their resilience and extended life cycle, microplastic particles (less than 5000 μm in size) have been in focus during the last decade. However, in the treated communal wastewater, a variety of anthropogenic fibers, including natural cellulose-based and petroleum-based fibers are the predominant solid particles^10–12^.

Since the toxicological effects of natural and microplastic fibers on aquatic organisms are comparable in a number of ways^13–15^, it would be necessary to investigate the emission of microplastic and natural fibers, as well as man-made cellulose fibers into the rivers. Prior to the discussion of natural and microplastic fibers in Athey and Erde’s critical review paper^16^, Ladewig et al.^17^ highlighted the potential of natural fibers to act as vectors of toxic compounds.

On the basis of their origin and chemical composition, the fibers as potential contaminants can be categorized into the following four groups^18^:

- (1) Natural cellulose-based fibers, including seed fibers (cotton, kapok), bast fibers (jute, flax, hemp, ramie, kenaf), and leaf fibers (abaca, sisal, henequen).
- (2) Natural protein-based fibers (wool, silk, and hair fibers, e.g., angora, mohair, alpaca).
- (3) Man-made semisynthetic polymer-based, regenerated cellulose fibers (viscose, modal, and lyocell), regenerated protein fibers (casein, arachinzein), cellulose esters, alginate.
- (4) Man-made synthetic petroleum-based fibers (polyester, polyamide, polypropylene, etc.).

These fibers are released from different textiles during the washing process in households and laundries or originate from personal care products^19–27^. The amount of fibers discharged during washing depends on the model of the machine and its operational parameters (temperature, time), textile characteristics (fabric type, age), and quality and concentration of detergent ^24,28,29^. On applying the same operating duration and wash load, the mass of fibers released in a top-loading machine was considerably higher than a front-loading one. In addition to possessing a central agitator that could result in more mechanical action, the top-load model’s longer cycle duration and higher water consumption could explain the weakening of the fibers^30^. Le et al.^25^ summarized that the application of an aggressive detergent, an abrasive high-speed washing process, and a high temperature results in a greater release of microfibers, particularly in the case of new fabrics.

Apart from the aforementioned sources of fibers, we also need to factor in the presence of cellulose-based fibers originating from disintegrated toilet papers made from virgin wood pulp or recycled papers that comprise cellulose fibers with length and diameter of 1–3 mm and 10–50 µm, respectively^31,32^. Their occurrence in the treated wastewater strongly depends on the treatment method, especially the screening used in the pre-treatment process. Although the natural cellulose-based materials are biodegradable, our knowledge of their degradation rate during the biological treatment and thereafter in the aquatic environment is limited. Hurwitz et al.^33^ investigated the efficiency of cellulose degradation in aerobic treatment settings, with a three-day contact period and temperatures between 13 and 23 °C. They found that temperature plays a dominant role; when the temperature was raised by 10 degrees, the degradation efficiency rose from 6.7 to 87%.

Zambrano et al.^24^ were the first to point out the lack of information on the aquatic biodegradation of different natural and synthetic textile fibers. Applying the ISO standard method for ultimate aerobic biodegradability of plastic materials in the aqueous medium (ISO 14851:1999, 2005), they were able to determine that, after 243 days, the percentage of biodegradation for cotton yarns was 75.9 + 12.35%, rayon yarns was 62.21 + 13.29%, 50/50 polyester/cotton yarns was 39.76 + 3.52%, and polyester yarns was 4.05 + 0.75%. This indicates that, depending on the environmental conditions, the biodegradation of cellulose-based fibers might take up to six months or more. Similar observations were made by Royer et al.^34^ when studying the biodegradation of wood-based cellulose fibers. However, it should be noted that in the case of both natural and synthetic fibers, this time is sufficient to produce adverse effects and result in the mortality of aquatic organisms.

In their critical review, evaluating 21 WWTP studies, Iyare et al.^35^ concluded that, on average, 88% and 94% of microplastics can be eliminated in secondary and tertiary WWTPs, respectively. During the preliminary and primary treatments, 72% of these contaminants were eliminated on average. It indicates that the majority of microplastics are present in the sewage sludge and small particles (especially those smaller than 150 μm) are discharged in the wastewater effluent. Therefore, it can be anticipated that different natural, semi-synthetic, and synthetic fibers are transported from municipal WWTPs located at the bank of rivers into the aquatic environment. Once ingested, these contaminants can pose a threat to aquatic organisms, resulting in adverse effects such as intestinal damage, reduced ingestion, decreased spawning, slow or delayed growth, shortened lifespans, and abnormal or even lethal gene expression^36^. However, it should be noted that the effect of these fibers depends on a variety of factors, including their size, surface properties, adsorbed contaminants, life cycle, etc.^13,15,37^.

On the basis of literature data, we aimed to elucidate the subject of microfiber emission from a secondary WWTP into the Danube River by distinguishing between microfibers derived from cellulose and petroleum, in addition to other microplastic particles. The sampling was planned to explore the potential effects of seasonally varying water temperature and its influence on biological activity of microorganisms and the domestic washing practices of the population over time. To obtain reliable information on the geometrical parameters (length and diameter) and chemical composition, all fibers in the 10–5000 µm size range were collected by sieving and filtration, manually separated, and examined individually using stereo- and fluorescence microscopes as well as the transflection FTIR method in the mid-infrared region of 600–4000 cm^−1^. Due to this comprehensive but time-consuming sample preparation procedure, it is possible to prevent fiber overlaps and the overestimation of fiber concentrations that can occur in the case of automated scanning measurements of fibers at a selected resolution. The resultant data of the microfiber load can serve as the basis for the development and enhancement of technological parameters, which would aid in the reduction of microfiber emission from municipal WWTPs.

## Results and discussion

### Occurrence of solid particles according to shape

**Figure 1:**
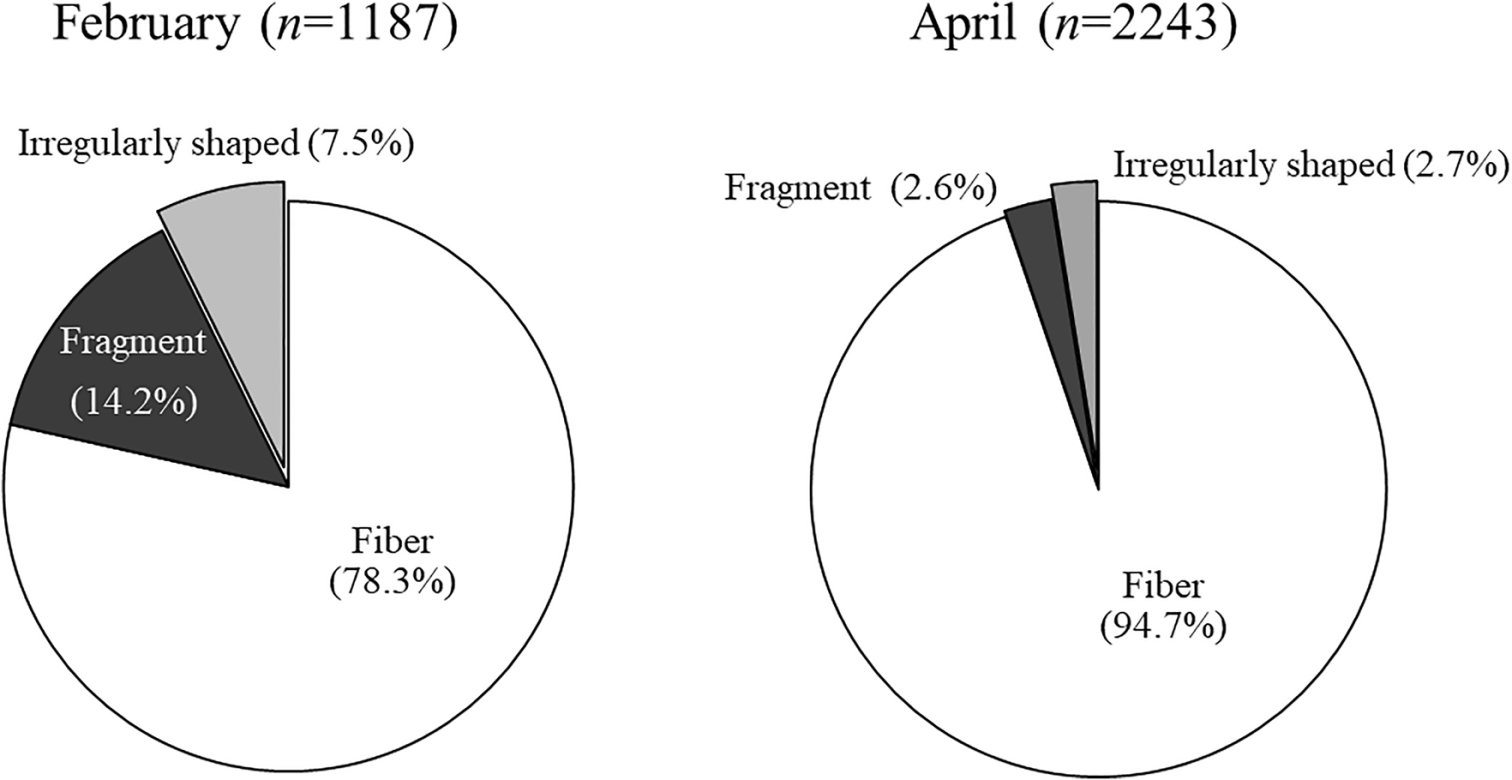
Proportion of fibers, fragments, and irregularly shaped particles in the 1187 and 2243 solid particles collected from 405 and 410 L of effluent sampled during the 7th (in February) and 16th (in April) week of 2023.

During the sampling campaigns in February and April, 405 and 410 L of effluents were sieved and filtered, respectively. The particles in the size range of 10 and 5000 µm were categorized according to their shape into fibers, fragments, and irregularly shaped particles. As illustrated in Fig. 1. Fibers were the predominant solids in both February and April, with 78.3 and 94.7% of the total, respectively. The total concentration of solid particles and, within these, the fiber concentrations measured on different working days in February and April are listed in Table 1. It can be established that both the total concentration of solids and the fiber concentration were nearly twice as high in April than in February; however, no pattern was noted with respect to the working days on which the measured data would indicate the association with household laundry. During February and April, the solid particle concentration fluctuated between 2.27–3.67 and 4.45–6.97 items/L, respectively. Similarly, the fiber concentrations in the effluent varied within the range of 1.88–2.84 and 4.25–6.79 items/L, respectively. The increased concentration of particles and fibers in April can be explained by the quick rinse of the about 3300 km long canal system remobilizing the deposited sludge and fibers by heavy rain (see Fig. S1.). It means the dilution effect of rainwater was overcompensated by this phenomenon.

**Table 1:** Volume of filtered effluents collected on different workdays and times during the 7th (in February) and 16th (in April) week of 2023.

Sampling date	Volume of filtered effluent (L)	Total numbers of counted particles	Fibers	Proportions of cellulose-based fibers (%)	Fragments	Irregularly shaped, particles	Concentration of solid particles items/L	Concentration of fibers items/L
7th weeks, February 2023								
Monday	140	515	398	73	74	43	3.67	2.84
8 am								
Wednesday 8 pm	123	349	265	53	56	28	2.83	2.15
Friday	142	323	267	66	39	17	2.27r	1.88
1 pm								
**Total**	405	1187	930		169	88	2.93	2.29
16th weeks, April 2023								
Monday	125	620	558	84	45	17	4.96	4.46
8 am								
Wednesday	140	977	951	91	9	17	6.97	6.79
8 pm								
Friday	145	646	617	84	6	23	4.45	4.25
1 pm								
**Total**	410	2243	2126		60	57	5.47	5.18

Total number of counted solid particles in the size range of 10–5000 µm, the number of fibers, fragments, and irregularly shaped particles, as well as the concentration of solid particles and fibers (items/L).

Due to higher fiber concentrations in April, the TOC value of the effluents depicted an increase of 29% as compared to February (Table S1). In the literature, the concentrations of microplastic particles found in secondary WWTP effluent have primarily been documented. These values vary from 0.25 to 125 items/L^3,38–40^. Sol et al.^10^ determined the concentration of microplastic fibers and observed values ranging between 1–347 items/L. The fiber concentration data in our study, which includes both cellulose- and petroleum-based (microplastic) fibers, was lower than the values detected by Sol’s group. The deviations in the particle and fiber concentrations can be linked to the differing composition of the influent and different operational parameters of the WWTPs.

### Length and diameter of microfibers collected from the WWTP effluents

**Figure 2:**
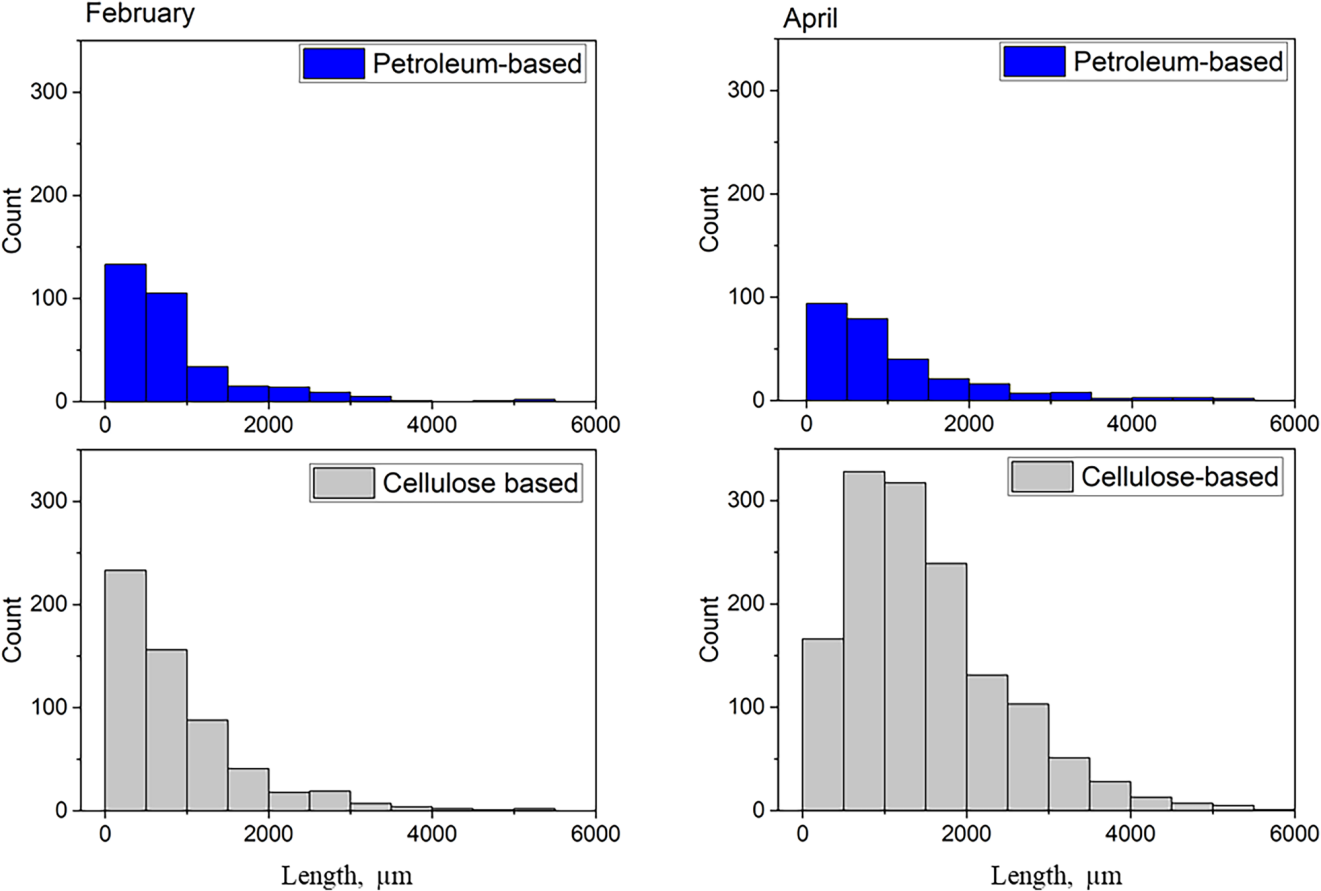
Distribution of cellulose-based and petroleum-based fibers collected in sampling campaigns during the 7th (in February) and 16th (in April) week of 2023 according to their length.

The length and diameter of fibers fixed individually on gel lifter covered carrier sheets were measured using a fluorescent microscope. Table 2 illustrates the median lengths and diameters of the cellulose- and petroleum-based fibers along with the length/diameter ratios. It is evident that the average values of both parameters were higher in April compared to February. This phenomenon is most likely the result of less efficient pre-screening. On the basis of the geometrical characterization of 3056 fibers, it can be stated that the median diameters and the median lengths of cellulose-based fibers were in all cases higher compared to petroleum-based fibers and the highest length/diameter ratio was observed also in case of cellulose-based fibers. As shown in Fig. 2, in February both fiber types had the highest occurrence in the length range of 250–500 µm. In April, the distribution function of petroleum-based fibers remained essentially the same, however, the maximum of cellulose-based fibers shifted in the range of 500–1500 µm. This shift in the length distribution indicates the emergence of a new fiber supply which can be linked to the aforementioned inefficient removal of toilet paper. It should be noted that the fiber dimensions fall in the range of previous studies focused on laundry^21,30,41^ and municipal wastewater facilities^6,12^.

**Table 2:** Median length and diameter of cellulose-based and petroleum-based fibers in effluent collected during the 7th (in February) and 16th (in April) week of 2023 and their length/diameter ratios.

Fiber type	Sampling time	Median length (µm)	Median diameter (µm)	Length/diameter ratio
Cellulose-based fibers (*n* = 2385)	February	650	21	30.9
	April	1250	29	43.1
Petroleum-based fibers *(n* = 671)	February	588	16	36.7
	April	620	20	31.0

### Proportion of cellulose-based and three petroleum-based microfibers in the effluents

**Figure 3:**
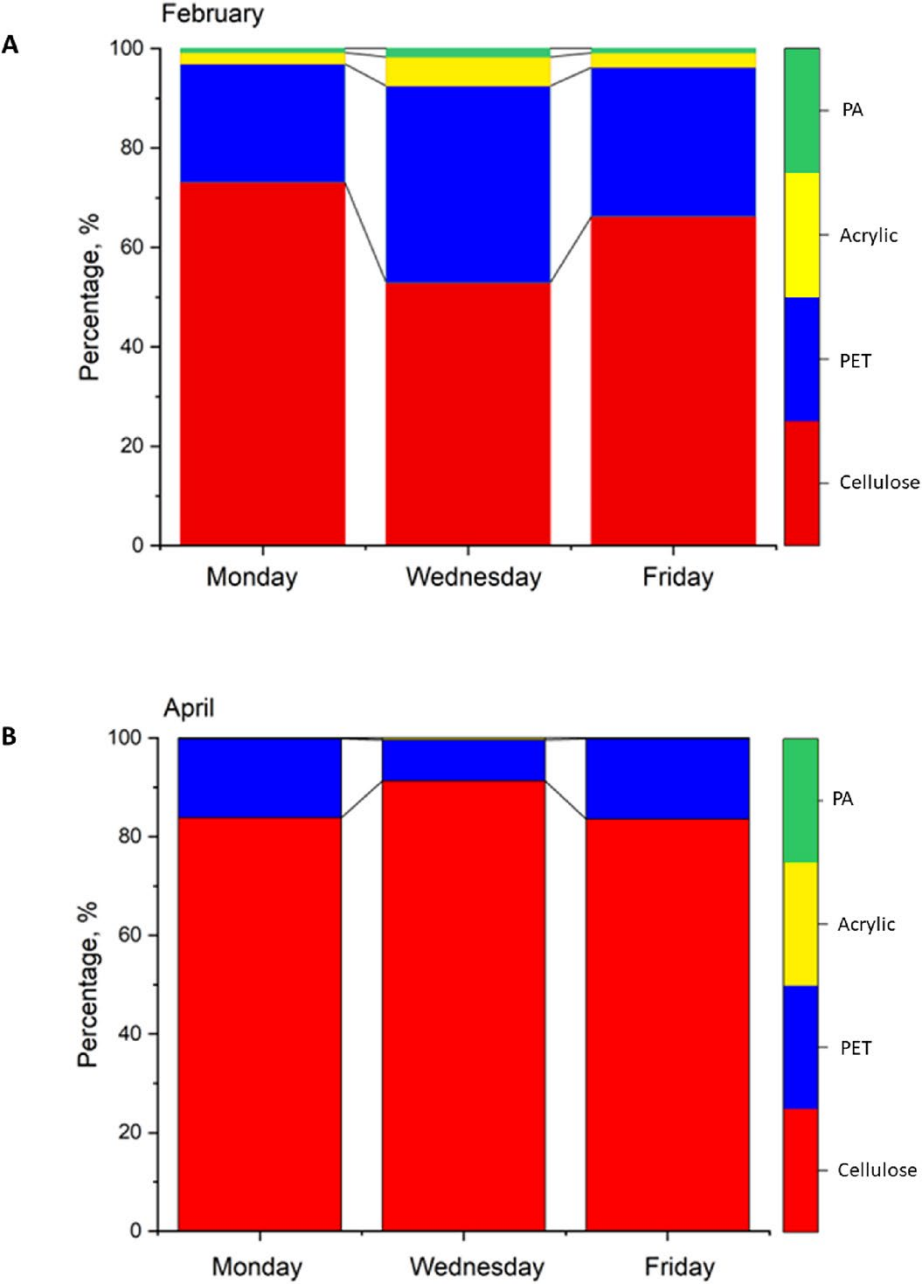
Proportion of cellulose-based fibers and three different types of petroleum-based fibers in the effluent collected on Monday, Wednesday and Friday during the 7th week (**A**) and 16th week (**B**) week of 2023.

For chemical identification of 3056 fibers, a FT-IR spectrometer in transflection mode was applied. Based on these investigations, the proportion of cellulose-based and three petroleum-based fibers was determined in the effluent samples. The proportions of cellulose-based fibers changed in the range of 53–73% and 84–91% in February and April, respectively (Fig. 3a,b. The higher occurrence of cellulose-based fibers in the effluent can be explained by the presence of disintegrating toilet paper made of wood-based materials or recycled papers^31,32^. On the basis of the reference spectra, polyester, acrylic, and polyamide (nylon) fibers were identified as petroleum-based fibers. These fibers were found in proportions of 27–47% in February and 5–16% in April. The fragments and irregularly shaped particles were identified as polyethylene, polyvinyl acetate, polyurethane, and polymethacrylate. In all cases, polyester predominated among petroleum-based fibers; its proportion was 70–73% in the winter and 90–97% in the spring. These results are harmonized with the Preferred Fiber and Materials Market Report^42^, which states that polyester fibers are produced globally in the greatest quantities and utilized in the manufacture of textiles. However, it is likely that the lower-density fibers were drawn to the water’s surface by the foam formation observed in April and were subsequently removed at a higher rate during the skimming of biologically treated wastewater. Consequently, during this time period, the proportion of polyester fibers-which have the highest density (1.3–1.4 g/cm^3^) among all petroleum-based fibers used in the manufacturing of apparel textiles-in the effluent increased.

### Daily microfiber transport to the Danube River

Based on the recorded amount of daily discharged effluent into the Danube River during our sampling days (Table 3) and the measured microfiber concentrations (Table 1), the daily emission of fibers was calculated and listed in Table 3. During the 7th and the 16th week of 2023, the number of microfibers emitted daily varied between 0.44–0.69 and 0.94–1.53 billion, respectively. Due to the relatively large water yield of the Danube River (yearly mean value of around 2300 m^3^/s), the effluent is considerably diluted by a factor of 500–1000, depending on the river’s actual water regime. Since the microfibers have small diameters 10–30 µm, they are easily ingestible, and therefore particularly dangerous for the zooplanktons and larger suspension-feeding organisms.

**Table 3:** Daily discharged effluent and fiber emission from the WWTP to the Danube River on different working days of the 7th (in February) and 16th (in April) week of 2023.

		Discharged effluent (m^3^)	Fiber concentration (items/m^3^)	Emitted fibers/day
February	Monday	219 885	2.840	624,473,400
	Wednesday	223 928	2.150	481,445,200
	Friday	221 954	1.880	417,273,520
April	Monday	235 869	4.460	1,051,975,740
	Wednesday	232 181	6.790	1,576,508,990
	Friday	214 579	4.250	911,960,750

It should be noted as a drawback that using sieves to separate solid particles forming size fractions from effluent that largely consists of fibers and sticky organic matter, is an ineffective method. The shorter fibers and the particles smaller than the mesh size are retained by the sticky organic matter deposited on the steel fibers of sieves. As a result, during our experimental work, we detected, counted, and characterized all fibers/particles with a size larger than 10 µm.

## Conclusion

The daily measured microfiber concentration in the effluent and the microfiber emission to the Danube River in April were more than twice higher than in February. Since the daily average amounts of treated wastewater (221,922 m^3^ in February and 227,543 m^3^ in April) were practically the same, the observed deviation in microfiber concentration can be attributed to the changes in the following factors: higher water temperature and corresponding higher biological activity of microorganisms, increased TOC content, and foam formation, as well as technological changes in the pre-filtration step (unclogging of the sieves in the primary treatment step) and remobilization of deposited sludge in the sewer network. Both in the winter and the spring samples the cellulose-based fibers were dominant representing 64 and 86% of the collected fibers, respectively. Considering the statistical data on the production of chemical fibers worldwide and the percentage of cellulose-based (8.38%) and synthetic (91.62%) fibers (Statista 2022), it is unforeseen that the concentration of cellulose-based fibers predominate in the effluent of the secondary WWTP investigated. This phenomenon can be attributed to the inefficient removal of cellulose-based fibers originating from toilet paper. In order to minimize the microfiber emission of municipal WWTPs and its adverse effects on the aquatic environment, it would be necessary to introduce modern technologies in four industrial domains:

1. The textile industry must develop new fabrics with lower shedding and reduced mass loss during the washing procedure by applying chemical finishing techniques.
2. The washing machine manufacturers must develop efficient filtration systems and optimize the agitation.
3. The toilet paper manufacturers need to develop new paper structures that reduce disintegration and facilitate the removal of larger pieces during the pretreatment process in WWTPs.
4. The WWTPs need to apply fine bar screens measuring 3 or 1 mm and fine mesh sieves with a size of 0.1 mm to enhance the recovery of cellulose-based materials from wastewater, as part of the valorization strategies of WWTPs in developed countries. Due to the lower cellulose content, the aeration demand and thereby the operating cost can be reduced.

## Materials and methods

### Study site and sampling

The investigated municipal WWTP located on the Csepel-Island in Budapest has an average daily capacity of 250,000 m^3^/ day. The wastewater from households, public institutions and industrial companies is transported to this facility through a nearly 3300 km long sewer network. It should be emphasized that the rainwater from the urban public area is also introduced to this network. This WWTP has a hydraulic retention period of 16–19 h and produces more than 50 tonnes of sewage sludge per day. Two sampling campaigns were carried out in the 7th (in February) and 16th (in April) week of 2023. The effluent samples were collected on Monday, Wednesday, and Friday during the period of 8–9 am, 7–8 pm, and 1–2 pm, respectively. The secondary treated wastewater was passed through a three-stage steel sieve system (Retsch AS 200, Germany) with mesh sizes of 710, 180, and 63 µm, driven only by gravitation. Owing to the clogging of the 63 µm sieve, the collected particles represent nearly 123–145 L of treated wastewater. The solid particles and dark brown organic matter from the sieves were washed into glass bottles with bi-distilled water and the suspensions passed through this finest sieve were also collected. The water temperature and pH were measured on-site and the total organic carbon (TOC) and total nitrogen (TN) were determined in the laboratory using a TOC/TN analyzer (Analytik Jena, Germany). The total phosphorous concentration of the effluent samples was measured with colorimetric EPA method 365.4, following the conversion of different phosphorous species to orthophosphate. These parameters are listed in Table S1.

### Sample preparation

The suspended particles/fibers and organic matter were separated from the water phase of the pre-concentrated samples using a MultiVac 600 unit (Rocker, New Taipei City, Taiwan) in an ESCO AC2- 4G8 laminar flow box (Airstream®, Singapore) applying Whatman glass fiber filters with diameter and pore size of 47 mm and 0.7 µm, respectively. Due to the clogging of these glass fiber filters, five filters were required in total to separate the solid particles/fibers originating from the 123–145 L treated wastewater. The dark brown organic matter was removed from the loaded wet filters by oxidation using particle-free hydrogen-peroxide (30%) treatment in Petri-dishes covered with aluminium foil and left at room temperature for 7 days. It is worth mentioning that the natural and man-made cellulose-based fibers, as well as the plastic fibers, lost less than 10% of their weight during the oxidation step ^43^. Because of this loss uncertainty, the mass concentration of solid particles and organic matter was not determined. Following the digestion process, the filters were dried at 80 °C for 12 h.

### Characterization and quantification of particles

Characterization and quantification of particles For the characterization of microfibers and microplastic particles according to size, shape, and number, the fibers and other particles were hand-picked individually from the dried glass fiber filters using a tweezer (Dumont Swiss-made 5/45 mode) and fixed on a B-17100 gel lifter (BVDA International B.V., Netherland) for visual inspection using a Carl Zeiss Stemi 508 stereomicroscope with a magnification of approximately 5-50x, an Olympus BX 61 fully motorized fluorescence microscope with a magnification of 40-1000x, and cell Sens Dimension 1.4 software. For their chemical identification, the fibers and particles were individually pressed onto steel plates to produce thin layers and investigated using the Bruker Vertex70 Fourier Transform Infrared (FT-IR) spectrometer equipped with a Hyperion 2000 FTIR-microscope and an Opus 7.2 Build: 7.2,139,1294 software. Each identification underwent 164 scans, with a minimum 10 × 10 µm area and 4 cm^−1^ resolution.

### Supplementary Information

Supplementary Information.

## Data Availability

Original experimental data is available from the corresponding author upon request.
