# Supplementary material for: Microfiber emission from a municipal wastewater treatment plant in Hungary
Source: Sci Rep. 2024 May 27;14:12041. doi: 10.1038/s41598-024-62817-2 (PMC11130252; doi:10.1038/s41598-024-62817-2)

Supplementary Material

Table S1 Physico-chemical parameters of effluents during the selected days of the 7^th^ and 16^th^ weeks 2023

| **Sampling date** | **Temperature (°C)** | **pH** | **TOC (mg/L)** | **TN (mg/L)** | **TP (mg/L)** |
| --- | --- | --- | --- | --- | --- |
| 13.02 Monday | 15.5 | 7.62 | 7.3 ± 0.5 | 12.5 ± 0.1 | 0.82 ± 0.04 |
| 15.02 Wednesday | 15.9 | 7.49 | 7.7 ± 0.1 | 12.5 ± 0.2 | 1.14 ± 0.06 |
| 17.02 Friday | 16.1 | 7.57 | 8.5 ± 0.7 | 11.8 ± 0.2 | 0.86 ± 0.05 |
| 17.04 Monday | 19.0 | 7.51 | 9.2 ± 0.1 | 9.4 ± 0.1 | 1.11 ± 0.09 |
| 19.04 Wednesday | 18.7 | 7.62 | 11.8 ± 0.2 | 13.5 ± 0.2 | 1.03 ± 0.08 |
| 21.04 Friday | 18.9 | 7.55 | 9.3 ± 0.1 | 10.8 ± 0,1 | 0.86 ± 0.04 |

Fig. S1 Daily precipitation in Budapest during February and April 2023 reported by Hungarian Meteorological Survey


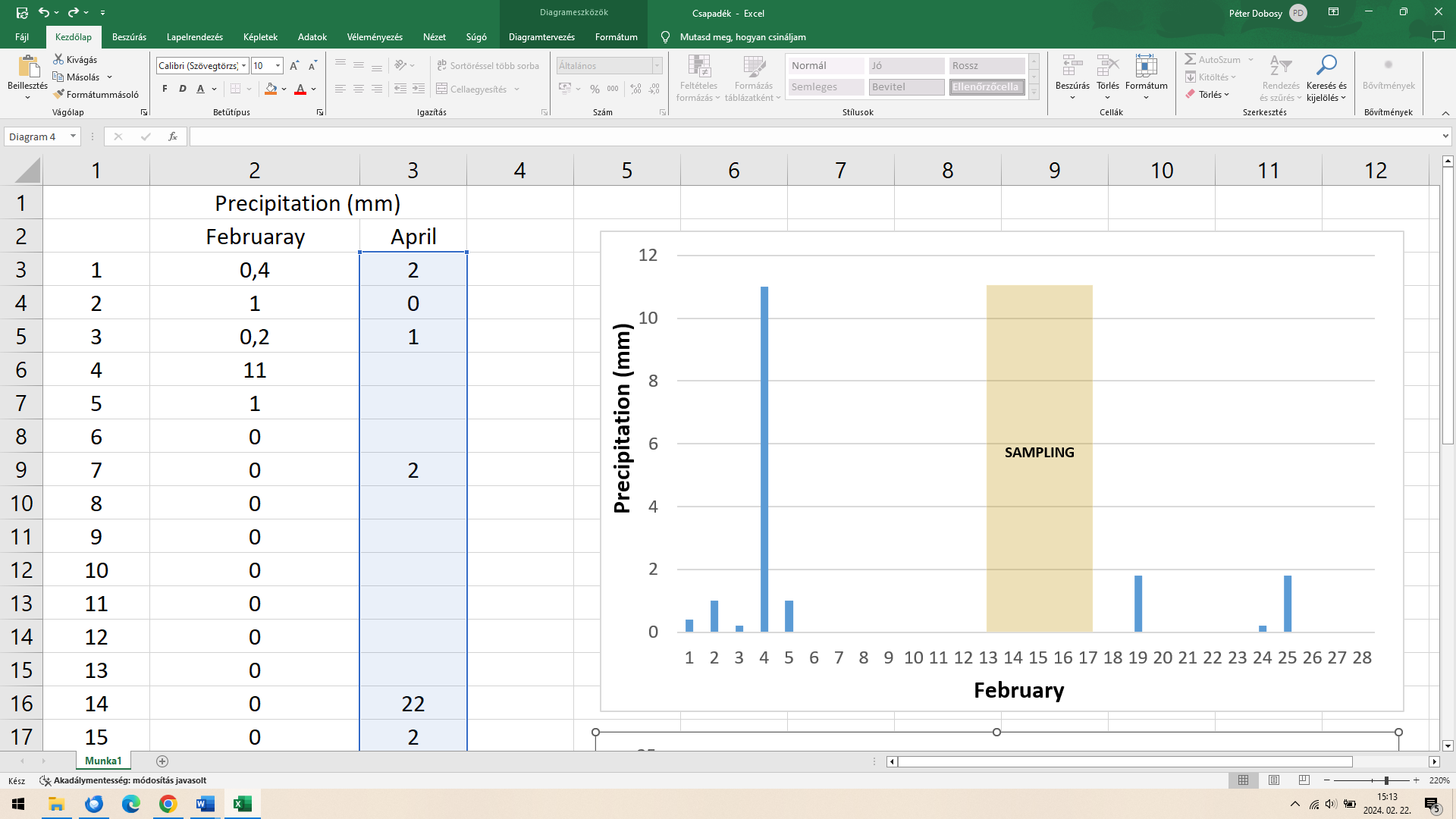


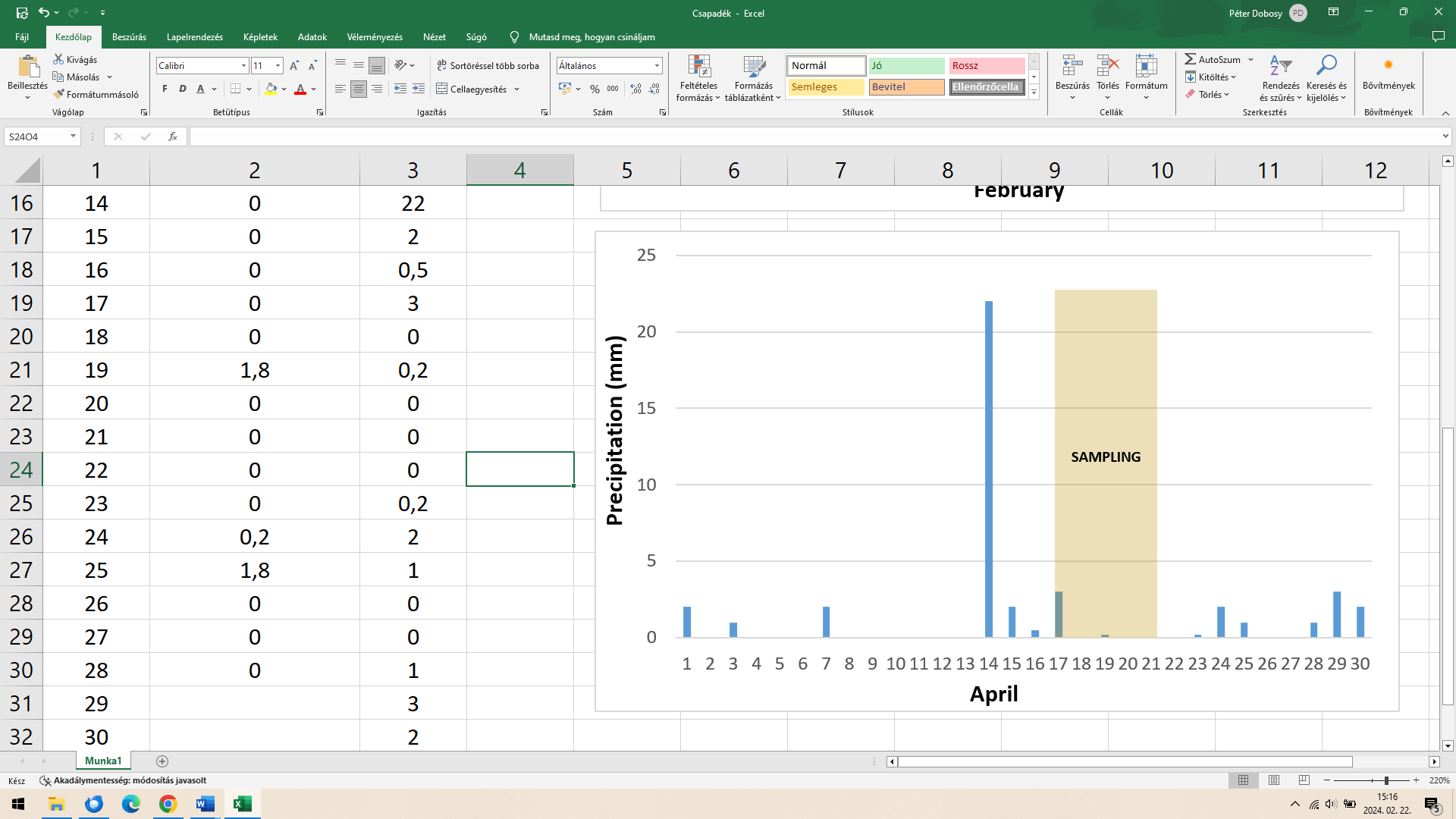

Supplement: Supplementary file 1 — Supplementary Information. [file 41598_2024_62817_MOESM1_ESM.docx]
